# CAREGIVING EXPERIENCES OF ADULT DAUGHTERS WITHOUT SIBLINGS IN UFA, RUSSIA

**DOI:** 10.1093/geroni/igad104.1138

**Published:** 2023-12-21

**Authors:** Polina Ermoshkina, Eva Kahana

**Affiliations:** Case Western Reserve University, Cleveland, Ohio, United States; Case Western Reserve University, Cleveland, Ohio, United States

## Abstract

This study is focused on the informal caregiving experiences of a unique group of women: only children of single mothers from Ufa, a metropolitan city in the Ural Mountains. Twenty middle-aged (M=40.75, SD=3.43), college-educated women participated in a two-hour, semi-structured interview. The average age of care recipients (mothers) was 65.3 (SD=3.85). Since the retirement age in Russia is 55 for women and 60 for men, all care recipients were retired. Inductive thematic analysis revealed three themes: incongruence of mothers’ needs with the microenvironment, aging in the “cement box,” and distrust of outsiders. Previous studies found that person-environment congruence is a determinant of older adults’ well-being. A discrepancy between older adults’ needs and preferences and environmental presses can become a source of chronic stress. This study found that both microenvironment (apartments) and larger environmental units (apartment buildings and neighborhoods) were incongruent with the needs of both older women and their caregivers. The majority of the housing stock in Russia is from the Khrushchev era: five-story buildings with no elevators or nine-story buildings with one small elevator. Older adults are trapped in their apartments due to steep stairs and a lack of elevators. Furthermore, adult daughters had to deal with conflicting cultural values and norms of care including the daughter’s duty and moral obligation to care for the aging parents and their own work-related and personal needs. Mothers’ dissatisfaction, in spite of daughters’ best efforts, resulted in unsatisfactory caregiving efforts and frustration by both caregivers and care receivers.

